# Temporal patterns of bacterial communities in the Billings Reservoir system

**DOI:** 10.1038/s41598-024-52432-6

**Published:** 2024-01-24

**Authors:** Marta Angela Marcondes, Rodrigo Pessôa, Alberto José da Silva Duarte, Patricia Bianca Clissa, Sabri Saeed Sanabani

**Affiliations:** 1 Post-Graduation Program in Translational Medicine, Department of Medicine, Federal University of São Paulo, São Paulo, 04021-001 Brazil; 2 Laboratory of Dermatology and Immunodeficiency, Department of Dermatology LIM 56, Faculty of Medicine, University of São Paulo, São Paulo, 05403-000 Brazil; 3 Laboratory of Immunopathology, Butantan Institute, São Paulo, 05503-900 Brazil; 4 Laboratory of Medical Investigation 03 (LIM03), Clinics Hospital, Faculty of Medicine, University of São Paulo, São Paulo, 05403-000 Brazil; 5 Laboratory of Dermatology and Immunodeficiency, LIM56/03, Instituto de Medicina Tropical de São Paulo, Faculdade de Medicina da Universidade de São Paulo, Av. Dr. Eneas de Carvalho Aguiar, 470 3º Andar, São Paulo, 05403 000 Brazil

**Keywords:** Water microbiology, Microbial ecology

## Abstract

In this study, high-throughput sequencing of 16S rRNA amplicons and predictive PICRUSt functional profiles were used to perform a comprehensive analysis of the temporal bacterial distribution and metabolic functions of 19 bimonthly samples collected from July 2019 to January 2020 in the surface water of Billings Reservoir, São Paulo. The results revealed that most of the bacterial 16S rRNA gene sequences belonged to *Cyanobacteria* and *Proteobacteria*, which accounted for more than 58% of the total bacterial abundance. Species richness and evenness indices were highest in surface water from summer samples (January 2020), followed by winter (July 2019) and spring samples (September and November 2019). Results also showed that the highest concentrations of sulfate (SO_4_^–2^), phosphate (P), ammonia (NH_3_), and nitrate (NO_3-_) were detected in November 2019 and January 2020 compared with samples collected in July and September 2019 (*P* < 0.05). Principal component analysis suggests that physicochemical factors such as pH, DO, temperature, and NH_3_ are the most important environmental factors influencing spatial and temporal variations in the community structure of bacterioplankton. At the genus level, 18.3% and 9.9% of OTUs in the July and September 2019 samples, respectively, were assigned to *Planktothrix*, while 14.4% and 20% of OTUs in the November 2019 and January 2020 samples, respectively, were assigned to *Microcystis*. In addition, PICRUSt metabolic analysis revealed increasing enrichment of genes in surface water associated with multiple metabolic processes rather than a single regulatory mechanism. This is the first study to examine the temporal dynamics of bacterioplankton and its function in Billings Reservoir during the winter, spring, and summer seasons. The study provides comprehensive reference information on the effects of an artificial habitat on the bacterioplankton community that can be used to interpret the results of studies to evaluate and set appropriate treatment targets.

## Introduction

Pollution of water resources remains a critical environmental concern across the globe. In developing countries, rapid urbanization, poor urban planning, and inadequate waste management exacerbate water quality deterioration, leading to increased microbial concentrations^1^. Bacterioplankton communities play a vital role in controlling energy flow and biogeochemical cycles within aquatic ecosystems. Their dynamics are subject to environmental factors such as total phosphorus^2^, pollution^3^, pH^4^, and dissolved oxygen (DO)^5^. Temporal variations in marine bacteria distribution and abundance suggest a seasonal influence on microbial diversity^6^. The biogeochemical cycling of nutrients by sediment bacteria is essential for maintaining the trophic state of water bodies^7–9^.

Understanding bacterioplankton diversity and its response to anthropogenic disturbances is key to detecting environmental changes in local ecosystems. Urbanization, for instance, has been linked to alterations in the physicochemical properties of aquatic environments, leading to biodiversity loss and reduced ecological function^10,11^. Changes in bacterioplankton community structure can significantly impact water quality, particularly due to the threat posed by toxin-producing *cyanobacteria*^12–15^. The interaction between *cyanobacteria* and heterotrophic bacteria influences the structure of bacterioplankton communities^16^, while bacteria serve as indicators of surface water contamination^17^. Pathogenic microbes and heavy metals also pose a significant threat to water quality, affecting human and animal health^18^.

Water properties fluctuate daily, with noticeable temporal and spatial variations^19,20^. Therefore, comprehensive monitoring of freshwater sources is crucial for maintaining suitable water quality. Reservoirs, which are limnologically a mix between a lake and a river, exhibit pronounced stratification in summer and potential reverse stratification in winter, affecting the distribution of chemicals and nutrients in the water^21,22^.

São Paulo, the largest urban area in Brazil, faces water stress with an annual water availability of less than 1,700 m3 per person in the São Paulo Metropolitan Region (SPMR)^23^. The Billings Reservoir, a significant freshwater source for the SPMR, serves multiple functions, including irrigation and flood control^24^. Despite its importance, the microbial community structure within the Billings Reservoir has been understudied since its construction in 1940^25^. Previous studies primarily focused on physicochemical variables and the presence of bacterial strains using culture-dependent methods^22,26–29^. Recent culture-independent studies conducted by our group revealed a higher prokaryotic diversity in Billings Reservoir compared to similar water systems and also a high abundance of *cyanobacteria*^24,30^. These bacteria are considered the most widespread photosynthetic organisms due to their global distribution and their significant ecological presence in nutrient-rich habitats, which often lead to the formation of blooms^31^. Cyanobacterial blooms are considered harmful to aquatic ecosystems as they are able to alter critical ecological parameters such as dissolved oxygen concentration, pH balance and light availability. Most species contributing to these blooms are capable of synthesizing cyanotoxins, leading to their classification as harmful cyanobacterial blooms^31^. The presence of cyanotoxins at Billings Reservoir was also shown by additional studies^21,25,32^. It is possible that nutrient inputs from agriculture and a rise in water temperature are among the triggers of the algal bloom in the reservoir, but this needs to be confirmed by further investigations. Without a complete phytoplankton succession series, however, the data are not yet sufficient to reveal the ecological processes of the algal bloom.

Therefore, this study involved bi-monthly sampling to examine the phytoplankton dynamics in the surface waters of Billings Reservoir, spanning from June 2019 to January 2020. The study hypothesizes that the Billings Reservoir exhibits significant seasonal shifts in phytoplankton composition due to environmental factors, with species displaying distinct adaptive responses to optimize survival and dominance in fluctuating conditions. The objective is to report on seasonal phytoplankton composition shifts and their adaptive responses to environmental conditions. The findings from this study offer a preliminary assessment of the unique ecological conditions formed by the seasonal activities within the reservoir.

## Material and methods

### Study area and sampling

**Figure 1:**
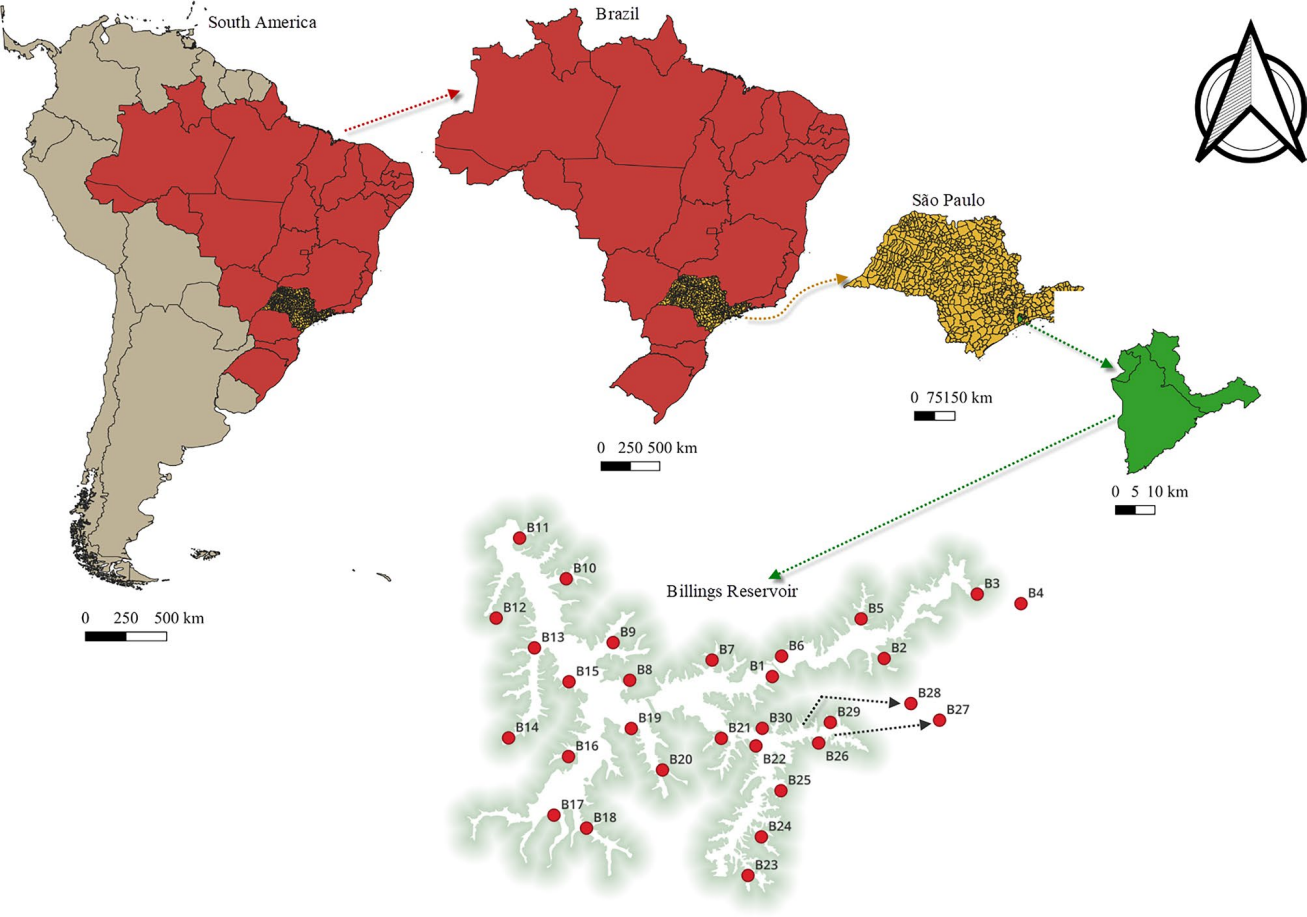
Map showing sampling site locations in the Billings Reservoir in São Paulo. The map was generated using QGIS 3.22.0 (Redlands, CA, USA).

Located in southeastern São Paulo, Billings Reservoir is fed by ten main tributaries, namely Rio Grande, Ribeirão Pires, Rio Pequeno, Rio Pedra Branca, Rio Taquacetuba, Ribeirão Bororé, Ribeirão Cocaia, Ribeirão Guacuri, Córrego Grota Funda, and Córrego Alvarenga. This reservoir is classified in the II class of Resolution 357 of the Brazilian Environmental Council (CONAMA), pursuant to Decree 10.755/77. The tree-shaped Billings Reservoir was built in 1925 during the water crisis as one of the solutions to minimize the impact of the crisis on public supply. Billings Reservoir is the largest water storage reservoir in the SPMR. It covers 127 km^2^ in six counties and has a total volume of 1228.7 × 10^6^ m^3^ with a water surface area of 10,814.20 ha and a maximum depth of 18 m^33–35^. It includes eight arms (or subregions): Rio Grande, Rio Pequeno, Rio Capivari, Rio Pedra Branca, Taquacetuba, Bororé, Cocaia, and Alvarenga. The reservoir's water quality is poor because the Pinheiro River, Brazil's most polluted aquatic ecosystem, is diverted into the reservoir to increase electricity generation (by water pumping) at the Henry Borden Power Plant on the São Paulo coast^27,30,36^. Since 1922, however, such pumping has been permitted only in the event of a flood in the capital city^30,37^. Currently, the reservoir is used as a water source, for fishing, navigation, recreation and hydroelectric power generation^38^. Approximately 250 ml of surface water (10 cm depth) was collected in triplicate at 30 locations approximately 18 km apart along the reservoir using a 5-L van spike sampler. Samples were collected in July 2019 (winter month), September 2019 (spring month), November 2019 (hot spring month), and January 2020 (summer month). At the beginning of the study, the geographic coordinates of the collection sites were recorded to ensure that subsequent water samples and measurements were taken on water from specific locations. Collected samples were stored in cool boxes at 4 °C in the dark and transported to the laboratory, where they were immediately processed as previously described^24,30^. The number of sites on the reservoir and their locations are shown in Fig. 1.

### Physicochemical analysis

Samples collected bimonthly were analyzed on site using a multiparameter water meter (YSI, USA) to determine the main physicochemical parameters, namely turbidity, temperature (Temp), dissolved oxygen (DO), and pH. Concentrations of nitrate (NO_3-_), sulfate (SO_4_^2−^), phosphate (PO_4_^3−^ (designated here as “P”) and ammonia (NH_3_) were analyzed from another subsample (250 ml) in the laboratory according to standard procedures (CONAMA 2005). These physicochemical parameters were frequently used to evaluate the ecological status of the reservoir according to the Brazilian standard (Order of the Minister of Environment, 20005).

### DNA extraction, library preparation, and sequencing

DNA was extracted from approximately 600 µl of each concentrated water sample by centrifugation of the surface water samples as detailed in our recently published study^24^. The PowerSoil DNA isolation kit (Mobio Laboratories Inc., Carlsbad, CA, USA) was used according to the manufacturer's protocol. DNA quality was checked by 1% (w/v) agarose gel electrophoresis, and the amount was determined using a Qubit 2.0 fluorometer (Life Technologies: Carlsbad, CA, USA). The extracted DNA was stored at -20 °C until further processing. PCR of the V3-V4 variable region of the 16S rRNA gene was performed using the Bakt_341F/Bakt_805R^39^, as previously described^40^. The amplicons of all samples were purified using a PCR clean-up kit (D4014, Zymo research) and then used as DNA template for a second round of PCR to index the DNA and prepare libraries as described previously^41,42^. The purified indexed libraries were pooled in equimolar amounts, diluted to four nM, and finally loaded onto an Illumina MiSeq cartridge for paired-end 300 sequencing.

### Data analysis pipeline and statistical analysis

Processing raw reads started with quality check and filtering of low quality (< Q25) reads by Trimmomatic ver. 0.32^43^. After QC pass, paired-end sequence data were merged together using fastq_mergepairs command of VSEARCH version 2.13.4^44^ with default parameters. Primers were then trimmed with the alignment algorithm of Myers and Miller^45^ at a similarity cut off of 0.8. Non-specific amplicons that do not encode 16S rRNA were detected by nhmmer^46^ in HMMER software package ver. 3.2.1 with hmm profiles. Unique reads were extracted and redundant reads were clustered with the unique reads by derep_fulllength command of VSEARCH^44^. The EzBioCloud 16S rRNA database^47^ was used for taxonomic assignment using usearch_global command of VSEARCH^44^ followed by more precise pairwise alignment^45^. Chimeric reads were filtered on reads with < 97% similarity by reference based chimeric detection using UCHIME algorithm^48^ and the non-chimeric 16S rRNA database from EzBioCloud. After chimeric filtering, reads that are not identified to the species level (with < 97% similarity) in the EzBioCloud database were compiled and cluster_fast command^44^ was used to perform de-novo clustering to generate additional OTUs. Following chimeric filtering, reads that could not be identified to the species level (with < 97% similarity) in the EzBioCloud database were compiled, and the cluster_fast command^44^ was used to perform de novo clustering to generate additional OTUs. Finally, OTUs with single reads (singletons) are omitted from further analysis. The secondary analysis which includes diversity calculation and biomarker discovery was conducted by in-house programs of Chunlab, Inc (Seoul, South Korea). The alpha diversity indices (ACE^49^, Chao1^50^, Jackknife^51^, Shannon^52^, NPShannon^53^, Simpson^52^ and Phylogenetic diversity^54^), rarefaction curves^55^, rank abundance curves^56^ are estimated. To visualize the sample differences, beta diversity distances were calculated by Jensen–Shannon algorithm^57^. PERMANOVA test was used to determine significant differences in beta diversity. With functional profiles that are predicted by LEfSe^58^ and Kruskal–Wallis H Test^59^). All analytics mentioned above were performed in EzBioCloud 16S-based Microbiome Taxonomic Profiling (MTP), which is a ChunLab’s bioinformatics cloud platform that automatically process the uploaded *fastq* data which are converted to MTP data unit. An MTP represents single metagenomic or microbiome sample.

Comparison of differences between water samples was performed using the one-way analysis of variance test (ANOVA). Samples with significant differences were subjected to the Turkey post-hoc test to determine which of the variables were statistically significant. To determine the causes of bacterial community dominance, principal component analysis (PCA) in Past4.07b software was used to examine the correlation between bacterial communities and environmental variables.

The map was created using ArcGIS software, version 10.2, licensed (ESRI, Redlands, California, USA). The administrative shapefiles used to create the map were obtained from the open-access domain of DataGEO: http://datageo.ambiente.sp.gov.br/. To display the study sites on the map, Global Positioning System (GPS) coordinates for the study sites were converted to shapefiles that were combined with the administrative shapefiles for the sites.

## Results

### Variation of water physicochemical properties

**Figure 2:**
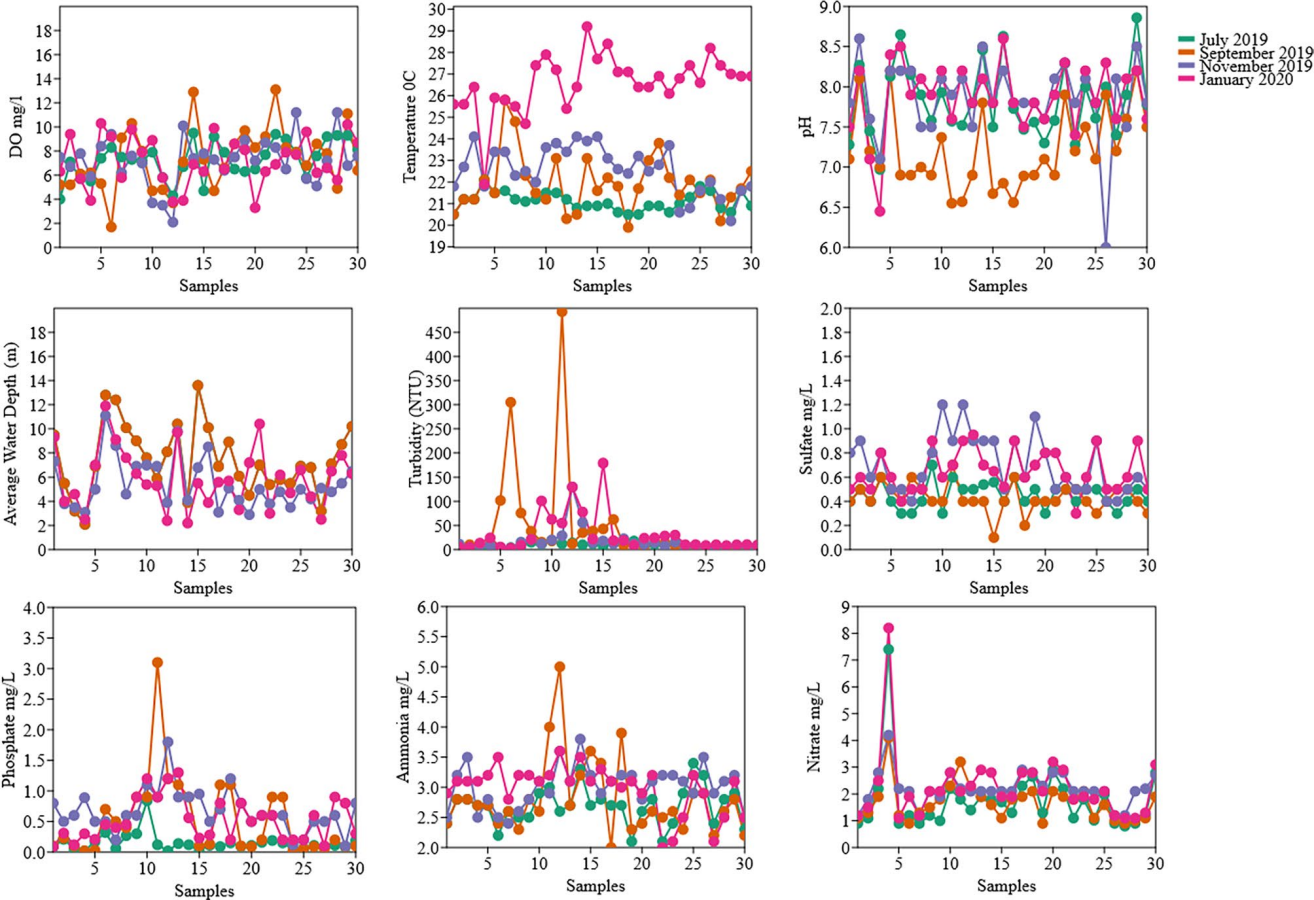
Seasonal variation of physicochemical properties of surface water samples from different sites of Billings reservoir during the study period (July 2019–January 2020).

**Figure 3:**
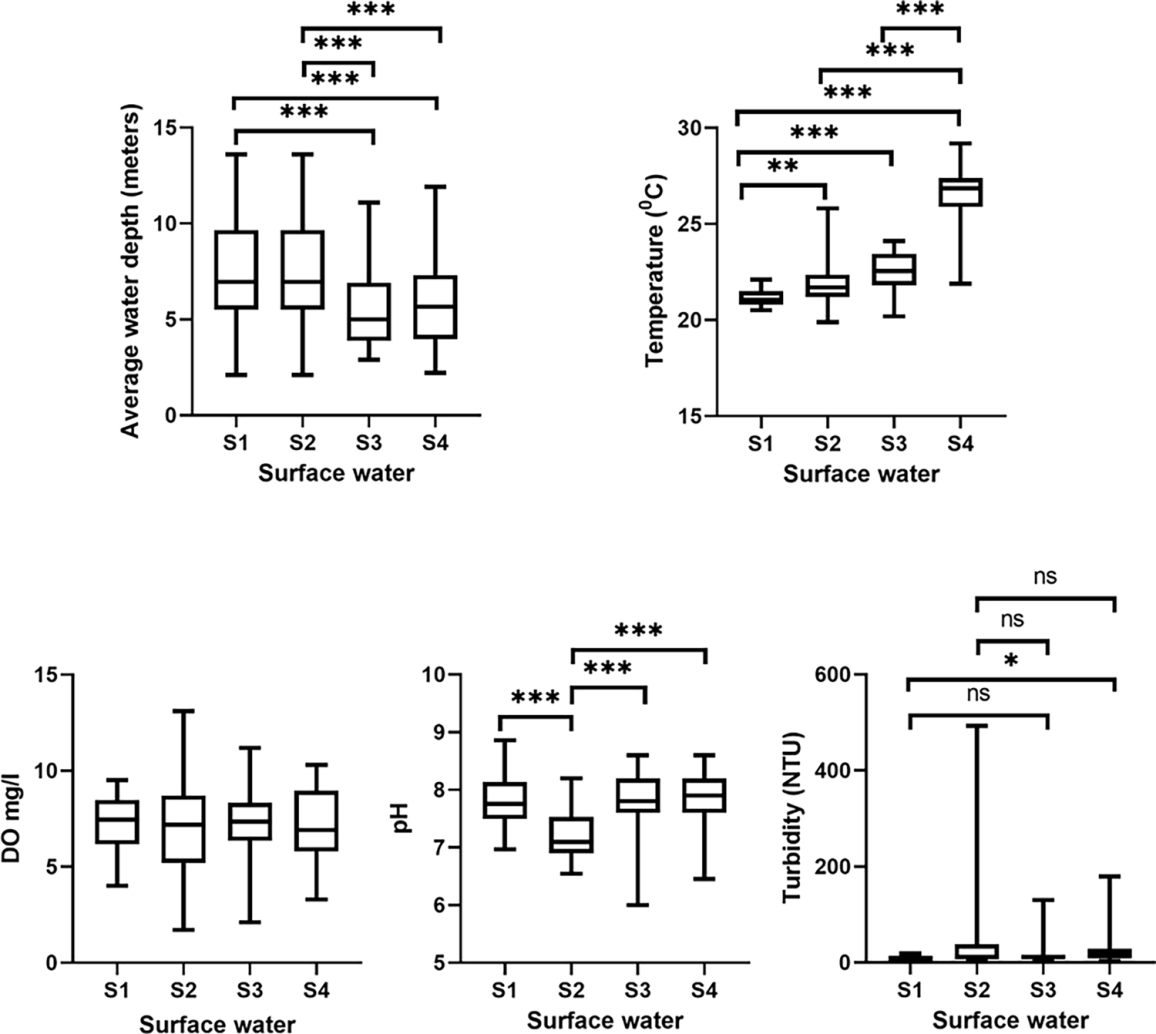
Boxplot showing variation of physicochemical parameters (average water depth, temperature, dissolved oxygen (DO), pH, and turbidity) of surface water samples from different sites of Billings reservoir during the study period (July 2019–January 2020).

**Figure 4:**
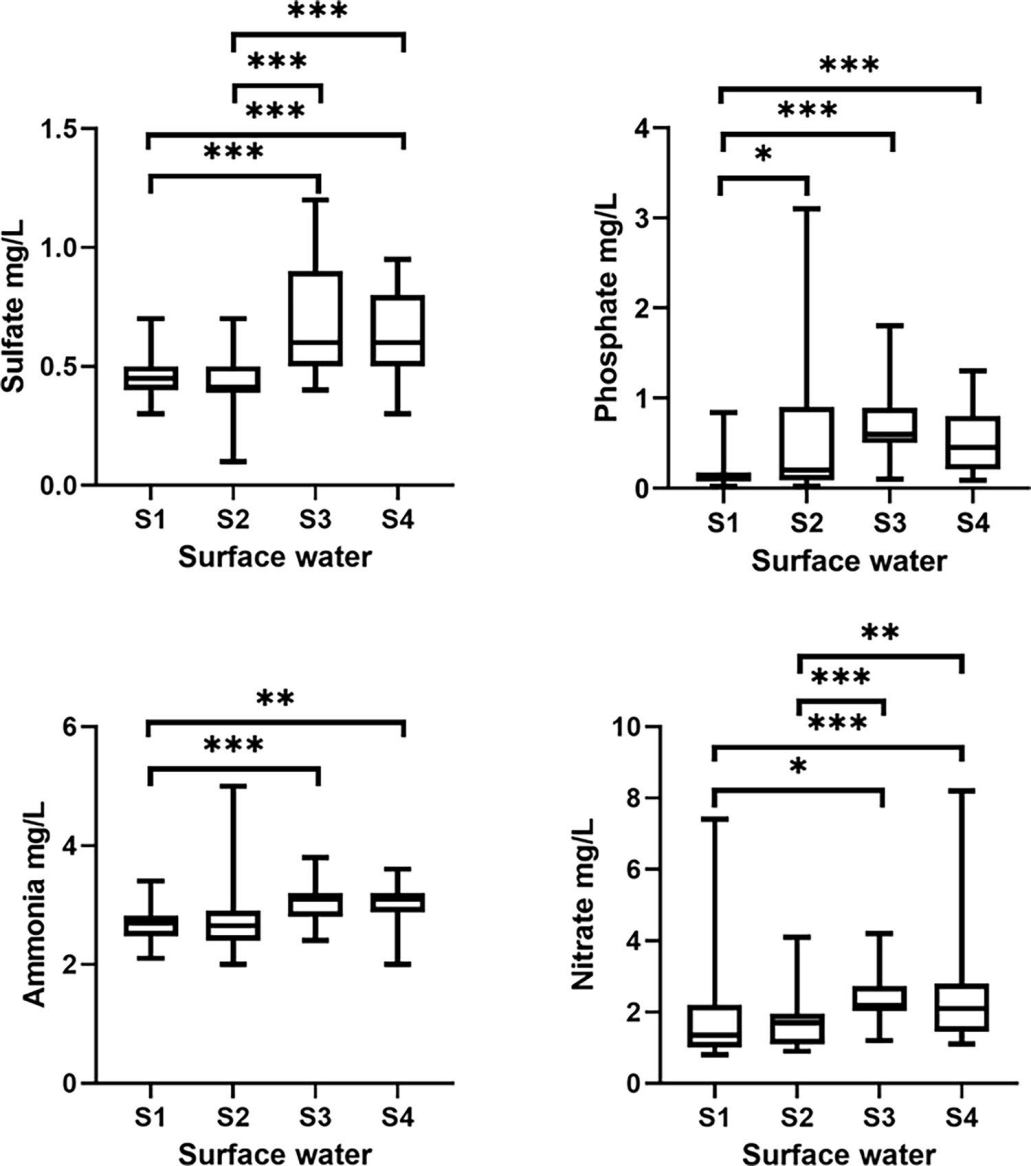
Boxplot showing variation of physicochemical parameters (sulfate (SO_4_^–2^), phosphate (P), ammonia (NH_3_), and nitrate (NO_3_)) of surface water samples from different sites of Billings reservoir during the study period (July 2019–January 2020).

To obtain information on the diversity and composition of the bacterioplankton community in the surface waters of Billings Reservoir, 30 sites along the reservoir were sampled bimonthly. Significant variations in physicochemical parameters were observed during the study period (Fig. 2**)**. For example, the average water depth or level of the reservoir decreased from 7.74 m in July and September (winter season) to 5.47 m in November 2019 and to 5.89 m in January 2020 (dry season), as shown in Fig. 3. In addition, the highest median temperature of 26.85 °C was observed in January (range 21.9–29.20 °C). The lowest and highest water temperatures were recorded at sites B14 (minimum of 20.90 °C in July 2019 and maximum of 27.70 °C in January 2020) and B18 (minimum of 19.90 °C in September 2019 and maximum of 27.10 °C in January 2020). No significant differences in DO were observed over time (Fig. 3). Site B06 showed the greatest variation at DO with the highest value of 9.4 mg/L in November 2019 and the lowest of 1.7 mg/L in September 2019 (Fig. 2). As shown in Fig. 3, pH was constant at most sites in July 2019, November 2019, and January 2020. A significant decrease in pH was observed for samples collected in September 2019. The same figure shows the greatest variation in pH in November 2019 (median 7.8; range 6–8.6) and January 2020 (median 7.9; range 6.45–8.6). The greatest variation in pH within the same site was observed at B26 (minimum of 6 in November 2019 and maximum of 8.3 in January 2020), B12 (minimum of 6.5 in September 2019 and maximum of 8.2 in November 2019), and B16 (minimum of 6.8 in September and maximum of 8.63 in July) (Fig. 2). Water turbidity varied over time, which was particularly evident in samples collected in January 2020 compared to samples collected in July 2019, namely B15, B12, and B09 (Figs. 2 and 3). The highest concentrations of SO_4_^2−^, P, NH_4+_ –N, and NO3^−^ were detected in November 2019 and January 2020 (Fig. 4).

The measured physicochemical water parameters were ordinated in a PCA (Fig. S1). Our interpretation on loading was considered significant if was greater than 0.40 or less than − 0.40 ^60^. In the July 2019 PCA, the first two principal components (PCs) accounted for 44.5% of the variability in the data set, with pH and DO along PC1 and water temperature and NH_4+_ –N along PC2 showing strong positive associations with water quality (Fig. S1a). However, it is important to consider that the increase in pH may be influenced by cyanobacterial blooms, which are known to elevate pH levels as a result of CO2 uptake during photosynthesis. Thus, the role of pH as a response variable rather than an explanatory one in the context of cyanobacterial abundance must be carefully considered when interpreting these PCA results. PC1 and PC2 explained 50.6% of the variability in the data set in the September 2019 samples, and phosphate concentrations and turbidity were most strongly and positively associated with PC1 (Fig. S1b). Along PC2, sulfate concentrations had a much greater loading than the eight other variables in this component. Analysis of PC1 and PC2 in the November 2019 samples explained 51.2% of the variance. As shown in Fig. S1c, PC1 showed a strong positive correlation with phosphate, sulfate, and turbidity, while PC2 was strongly associated with water depth. Individual loading analysis of the January 2020 samples showed that PC1 and PC2 explained 53.6% of the variability in the data set. Sulfate and nitrate concentrations were most strongly associated with PC1 and pH and phosphate concentrations were most strongly associated with PC2 (Fig. S1d). PC1 scores of water turbidity and nutrient variables followed similar patterns in bi-monthly samples collected from September 2019 to January 2020. DO Concentrations radiated in opposite directions, indicating a strong negative correlation of these variables. Algae were observed floating on the surface of the reservoir at some locations during the January 2020 sampling.

### Sequencing depth and diversity analysis

**Figure 5:**
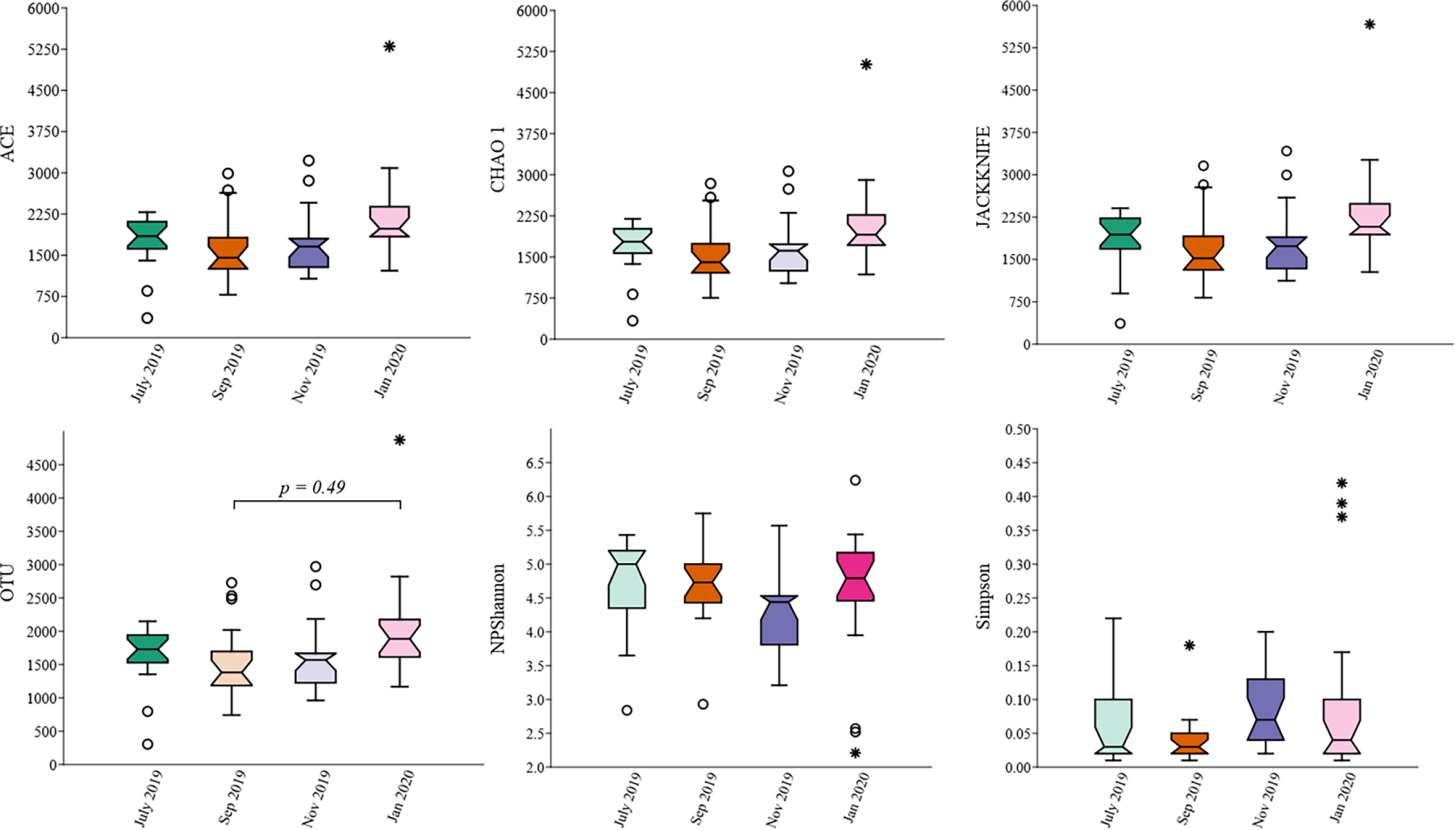
Alpha diversity indices comparing the four sample groups. No significant differences were found between the groups.

**Figure 6:**
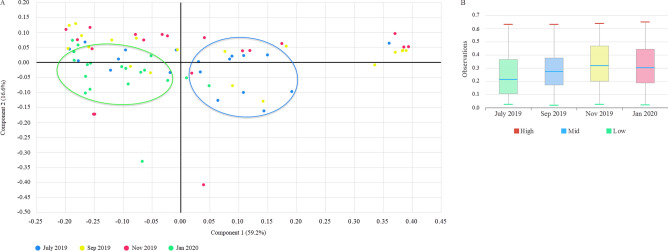
Beta diversity computed by **a** principal coordinate analysis (PCoA) and PERMANOVA test and **b** pairwise comparisons of the bacterioplankton communities in the surface water of Billings reservoir during the study period (July 2019–January 2020).

Of the 30 sites, 76 site-matched water samples (19 × 4 bimonthly samples) were successfully amplified and sequenced from surface layers and submitted for 16S rRNA analysis. Up to 100,000 MPS reads from each sample were uploaded, quality controlled, and profiled using the EzBioCloud tool. Trimming-based quality control removed 142,347 low-quality amplicons from all samples. The taxonomic approach detected and removed 243,108 and 1,629,579 non-target and chimera amplicons, respectively. The total quality assessment and trimming steps resulted in 4 945 059 valid reads from all samples considered for further analysis. On average, each sample or MTP was represented by 65,066 ± 7,604 valid reads. The average length of these reads was approximately 435 (± 17.22) nucleotides, with average minimum and maximum lengths of 409 (± 2.2) and 455 (± 2.3) nucleotides, respectively. Of the valid reads, 3,489,504 (70.6%) sequences were identified at the species level with a similarity cutoff of 97%. The average of Good's coverage estimator for OTUs in all MTPs was 99.6 (± 0.22), indicating that the diversity of bacterioplankton in all MTPs was adequately covered by the generated sequences. The number of observed OTUs determined in the water samples of the January 20 samples was higher than that of the September 19 samples (Bonferroni-corrected *p* = 0.049). When analyzing alpha diversity using the diversity indices ACE, Chao 1, Jackknife, NPShannon, or Simpson, we found no significant differences among the four sample groups (*p* > 0.05) (Fig. 5). For beta diversity, PERMANOVA analysis revealed significant differences in bacterioplankton structure between sampling period (Fig. 6a). Pairwise comparisons revealed significant differences in beta diversity between July 2019 samples compared to all other sampling sites (*p* < 0.001) and between Sep 2019 and Jan 2020 (*p* = 0.019) (Fig. 6b).

### Bacterial community identification at phylum and genus levels

**Figure 7:**
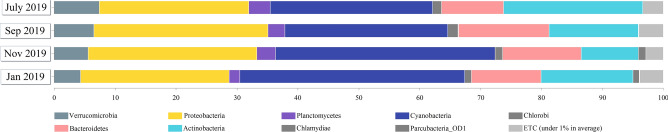
Phylum level composition of bacterioplankton communities within the surface water of billings reservoir during the study period (July 2019–January 2020).

**Figure 8:**
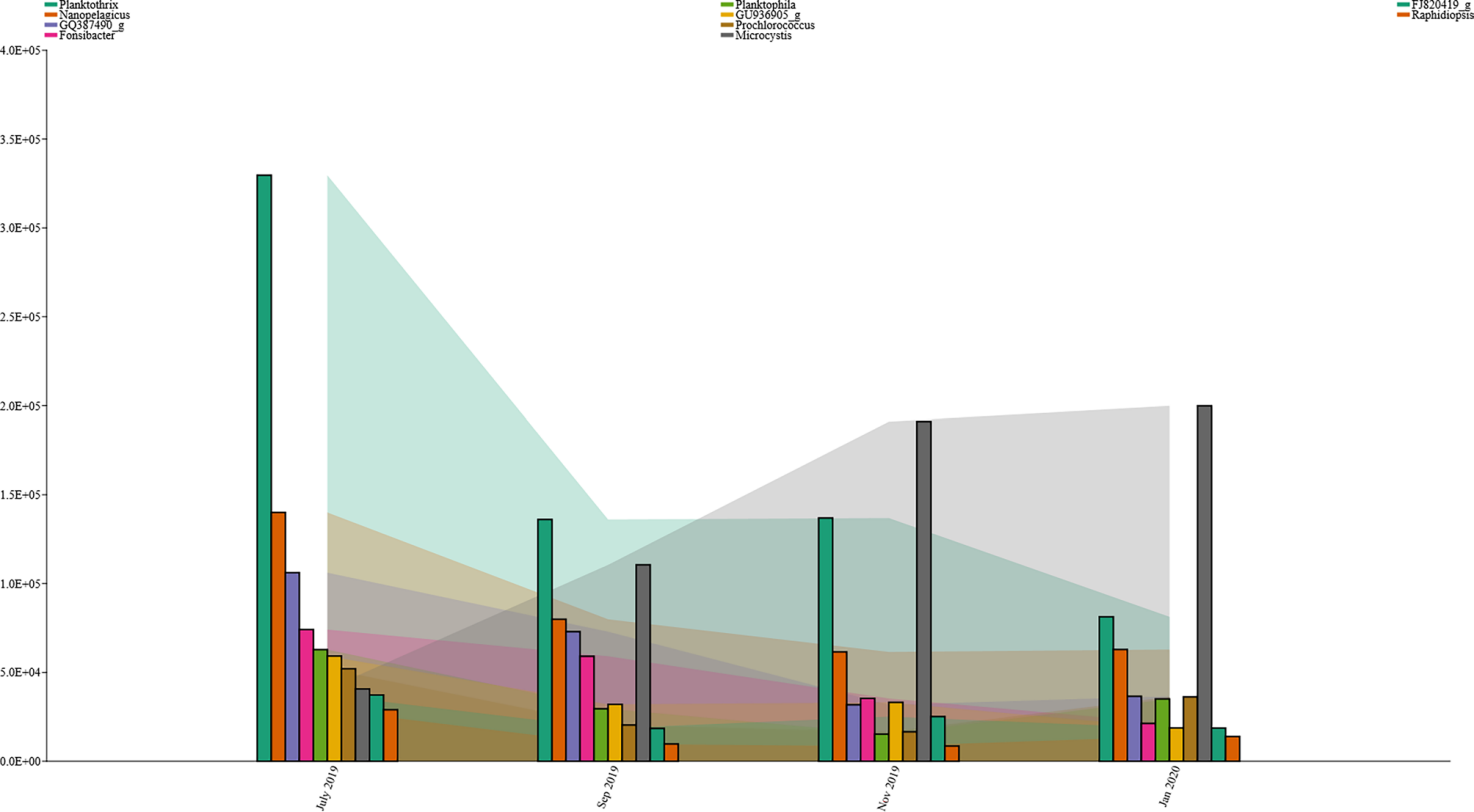
The distribution of the 10 most frequently detected bacterioplankton genera in all samples analyzed at four time points.

**Figure 9:**
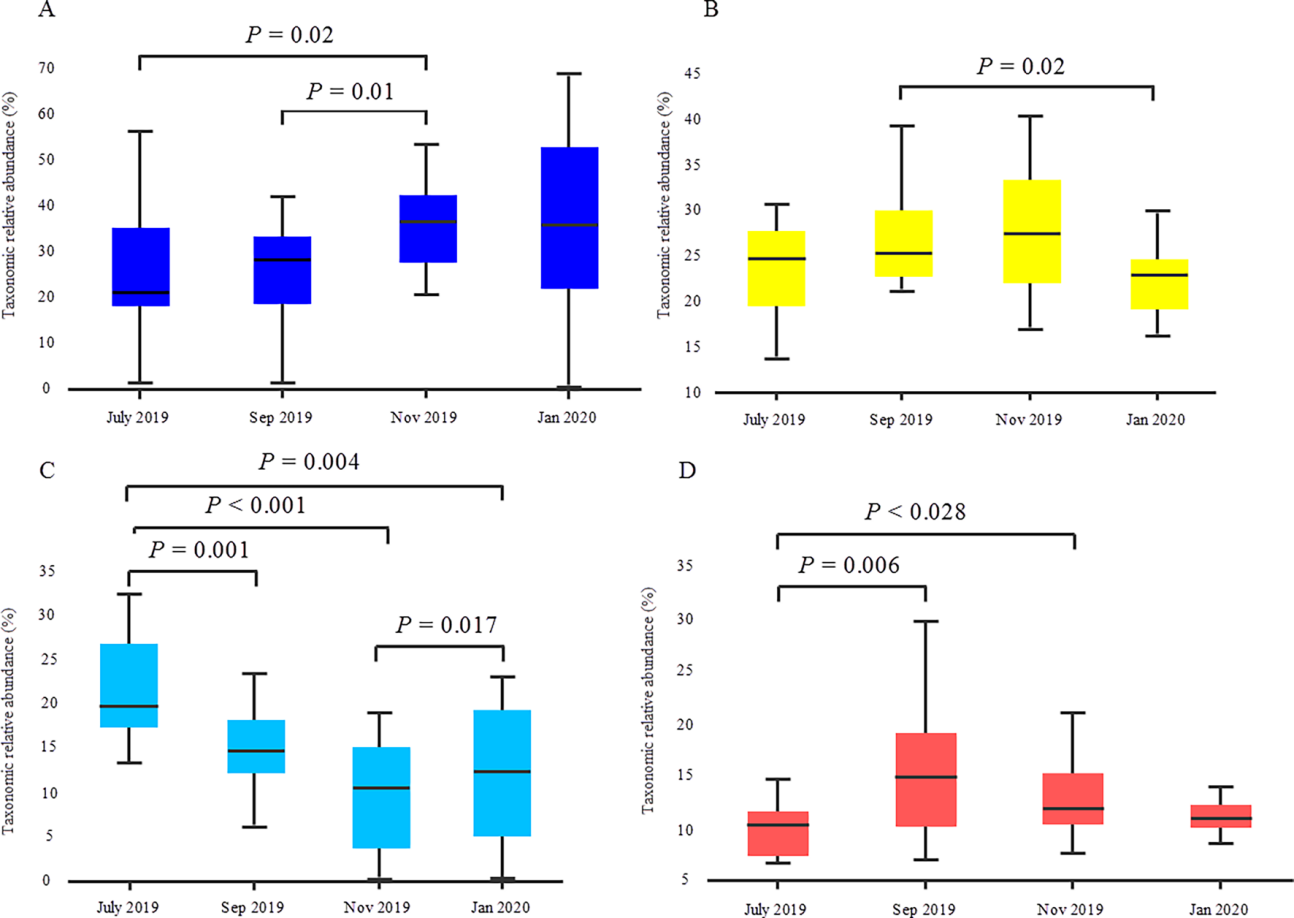
Boxplot showing the phylum relative abundance of the most abundant taxa of (**A)** *Cyanobacteria*, (**B)** *Proteobacteria*, (**C)** *Actinobacteria*, and (**D)** *Bacteroidetes* in four sample groups*.* Box borders represent the first and third quartiles, and the central lines represent the medians.

Sequences from all data sets of the four sample groups could be assigned to 48 phyla. The overall composition of bacterioplankton at the phylum level of all surface samples is shown in Fig. S2. Among these groups, *cyanobacteria* were the phyla with the most assigned sequences, averaging 31.75%. Other dominant bacterial phyla were *Proteobacteria* (26.5%), *Actinobacteria* (15.5%), *Bacteroidetes* (12.25%), *Verrucomicrobia* (6%), *Planctomycetes* (3%), and *Chlorobi* (1.25%) (Fig. 7). At the genus level, 18.3% and 9.9% of OTUs in the July and September 2019 samples, respectively, were assigned to the genus *Planktothrix*, while 14.4% and 20% of OTUs in the November 2019 and January 2020 samples, respectively, were assigned to the genus *Microcystis* within the family *Chroococcaceae*. The distribution of the 10 most frequently detected bacterioplankton genera in all samples analyzed at four time points is shown in Fig. 8. The relative abundance of *cyanobacteria* generally increased from July to September (26.7%) and increased in the following months to > 35% in November and January (Wilcoxon rank sum test, *p* < 0.05) (Fig. 9A). It is noteworthy that the different sampling sites showed different trends in the relative abundance of *cyanobacteria*. For example, in July, the lowest concentration of *cyanobacteria* was detected in sample B04, while the highest concentration was measured in sample B12 (Fig. S2). This scenario was also observed in January, when sample B04 had the lowest concentration of *cyanobacteria*. Strong concentrations of *cyanobacteria* were observed in all samples collected in November, with their relative abundance exceeding > 50% in sample B15 and > 20% as the lowest concentration in sample B29. A greater amount of *Proteobacteria* was observed in September 2019 than in January 20 (Wilcoxon rank sum test, *p* < 0.05) (Fig. 9B). At the same time, the relative abundance of *Actinobacteria* in the July 2019 samples was remarkably higher than the relative abundances calculated at other sampling times (Fig. 9C). In addition, the relative abundance of *Bacteroidetes* was significantly lower in the July 19 samples than in September 19 and November 19 (Fig. 9D). The abundance of the remaining bacterial phyla, including *Verrucomicrobia*, *Planctomycetes*, and *Chlorobi*, did not differ significantly in the bi-monthly samples.

In addition to the predominant phyla in Billings Reservoir, there were approximately 3.98% unclassified OTUs in the surface water, indicating as yet unidentified bacterial populations. The detailed distribution of these new bacteria by location and date of collection is shown in Fig. S3.

**Figure 10:**
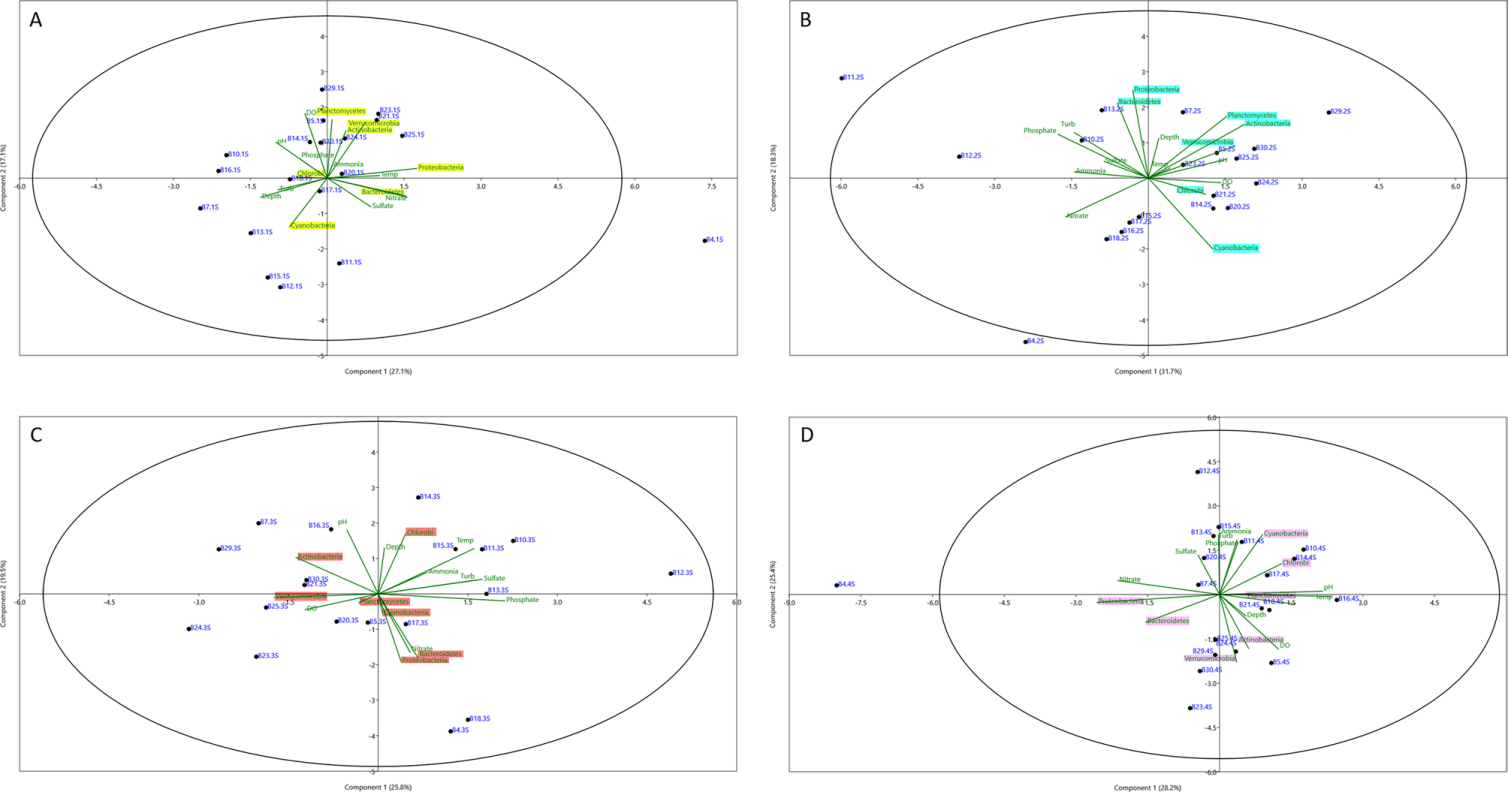
Principal component analysis (PCA) of the distribution of the 7 most abundant phyla (highlighted in color) relative to nine environmental variables in sample groups collected in **A** July 2019, **B** November 2019, and **D** January 2020. Samples are written in blue and indicated by a black dot.

We then investigated the relationships between physicochemical variables and bacterial composition of the seven major phyla. Analysis of the July 2019 samples showed that the abundance of *cyanobacteria* was positively related to water depth and turbidity, and that *Proteobacteria* and *Bacteroidetes* were strongly related to temperature and nitrate levels, respectively (Fig. 10A). The abundance of *Proteobacteria* was most strongly and positively associated with PC1, explaining 27% of the variance in this component. Along PC2, the concentrations of DO, the relative abundances of *Planctomycetes* and *Verrucomicrobia* had a much greater loading than the 13 other variables in this component. Analysis of PC1 and PC2 in the September 2019 samples explained 49.5% of the variance. PCA showed that the abundance of *Actinobacteria*, *Planctomycetes*, and *Verrucomicrobia* was most positively associated with pH and temperature (Fig. 10B) and that taxa belonging to *Proteobacteria* and *Bacteroidetes* had the strongest loadings on PC1. The first two principal components of the Nov-2019 samples explained 45.3% of the variability in the data set, with P concentrations most strongly and positively associated with PC1 and *Proteobacteria* abundance negatively associated with PC2. Figure 10C shows that *Proteobacteria*, *Bacteroidetes*, and *Cyanobacteria* were positively associated with nitrate concentration in water, while *Actinobacteria* were related to pH. The abundance of *Planctomycetes* and *Verrucomicrobia* was related to the concentrations of DO. Finally, PC1 and PC2 of Jan-2020 samples explained 53.6% of the variance, and the individual variable loadings showed that temperature and pH were the variables most positively associated with PC1, while *Proteobacteria* and *Verrucomicrobia* were negatively associated with PC1 and PC2, respectively. The PCA shown in Fig. 10D indicated that *Actinobacteria* and *Verrucomicrobia* were associated with the concentrations of DO and water depth, and that the abundance of *Planctomycetes* was affected by temperature and pH.

### Taxonomic biomarker discovery and functional analysis

**Figure 11:**
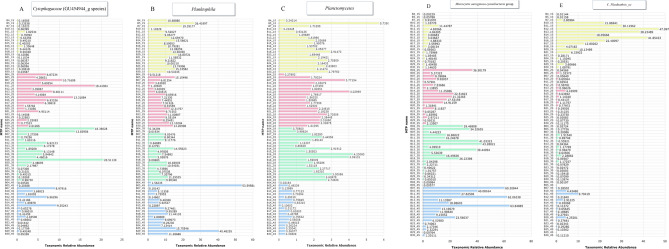
Linear discriminant analysis effect size (LEfSe) of the five most affected eukaryotic phytoplankton bacteria with an LDA score higher than 2.0 and P values less than 0.05 in all sample groups.

LEfSe analysis was performed on bimonthly samples using KEGG orthology (KO) of metabolic functions predicted by PICRUSt to assess taxonomic biomarkers. The Kruskal–Wallis method was used to examine grouped data with a significance of 0.05, and the statistically differentially distributed KO Ids were used for LDA model analysis, with relative frequency of significance assessed by an LDA threshold of 2.0. A total of 32 genera and 41 species were significantly more abundant in all paired comparisons between sampling dates (*p* value (FDR) < 0.05, LDA effect size ≥ 3.0) (Table S1). *Cytophagaceae* (GU454944_g species) and *Planktophila* were among the bacteria whose relative abundances differed significantly (Fig. 11A and B). For example, the September 19 and November 19 samples were characterized by a preponderance of *Cytophagaceae* (LDA score 4.37, *p* value (FDR) = 0.00036), while the July 19 bacteriome was characterized by a preponderance of *Planktophila* compared to the other sampling dates (LDA score 3.98, *p* value (FDR) = 0.004). LEfSe analysis also showed that *Planctomycetes* were significantly less abundant in the January 20 samples compared to the other sampling dates (LDA score 3.92, *p* value (FDR) < 0.0001) (Fig. 11C). The analysis also showed that the *Microcystis aeruginosa cyanobacteria* group was significantly more abundant in the January 20 samples (Fig. 11d *Microcystis aeruginosa cyanobacteria* group), while *C. Planktothrix_uc* species were detected significantly more often in the July 19 samples (Fig. 11C and E*. Planktothrix_uc*).

Analysis of the potential functions of the metagenomes between collection dates yielded a total of 5575 significant KO Ids for all paired comparisons between collection dates (*p* value (FDR) ≤ 0.05) (Table S2). Metabolic pathways (ko01100) was the most abundant functional gene category, followed by biosynthesis of secondary metabolites (ko01110), microbial metabolism in different environments (ko01120), two-component system (ko02020), ABC transporters (ko02010), biosynthesis of cofactors (ko01240), and carbon metabolism (ko01200).

## Discussion

In this study, we examined the spatiotemporal changes in bacteriological and physicochemical parameters over the course of a year in 19 bi-monthly surface water samples collected from Billings Reservoir. Genome sequencing of the 16 s rRNA gene was generated using the Illumina MiSeq platform and used to analyze variance in bacterial community structure.

### Variation in physicochemical parameters

The characteristics of the surface water from Billings Reservoir showed seasonal differences in physicochemical composition. For example, as expected, water surface temperature, an important abiotic factor affecting aquatic microbial communities, was on average 4.70 °C warmer during the summer months than during the winter months. A temperature fluctuation of this magnitude can greatly accelerate the growth rates of *cyanobacteria* and mesophilic bacteria^61–63^. Seasonal changes in temperature have also been reported as the principal factor driving alterations of the bacterial populations in freshwater Lake Taihu^64^ and in Pearl River Estuary sediments^65^. In addition to temperature fluctuations, we have found that the amount of nutrients in the reservoir changes with the seasons. This may have affected not only the bacterioplankton communities^66,67^ but also led to seasonal responses in the fish communities^68^. Our results are consistent with a previous study by Mankiewicz et al.^69^ who described the dynamics of *Microcystis* in the summer of five consecutive years at Sulejów Reservoir and concluded that optimal nutrient concentrations, high water retention, and temperature were the main causes of the prevalence of Cyanobacteria producing toxic microcystins. In other studies, the availability of nitrogen, not phosphate, was considered one of the most important factors in the dominance of toxic over nontoxic *Microcystis* populations, usually caused by gradual anthropogenic eutrophication^70,71^. In contrast, DO, which often affects bacterial communities in aquatic ecosystems^72^, does not vary significantly over the course of the year, so it was not considered to affect bacterioplankton communities in Billings surface water. Consistent with our results on the water surface from Billings reservoir, Pierangeli et al.^73^ recently found that DO and groundwater pH showed no statistical differences among sampling sites. According to our results, the water was neutral, with a relatively large increase in pH in September, the month of transition from the dry winter to the hot rainy season. All surface water samples collected from the reservoir, regardless of season, had an average pH of 7.6 and a range of 6.0 to 8.86, which is within the pH range determined by WHO for most natural waters. The higher pH than the upper limit of 8.5 could be due to abundant rainfalls which promotes leaching and dissolution of salts^74^ or to algal growth, which could be promoted by increased CO2 consumption during photosynthesis^75^. Our results also showed that the water level in the reservoir was lowest during the summer season due to increasing temperatures, increasing evaporation, and increased demand for human consumption. Many studies have shown that water depth affects the physicochemical composition of the water as well as the release of nutrients from the sediment and favors the growth of certain bacteria^76–79^.

### Bacterioplankton dynamics and community structure

As expected, the results showed that seasonal variations dominate the spatial patterns of bacterioplankton communities in the reservoir, as evidenced by the diversity indices and community composition. In particular, summer showed higher diversity indices and different bacterioplankton community composition than the other seasons. These differences in abundance could have been caused by significant seasonal variations in temperature and the availability of inorganic nutrients such as nitrogen in the reservoir.

The phytoplankton of Billings Reservoir showed that the *cyanobacteria* group quantitatively dominated the community structure, especially in summer. These results are consistent with those of other studies in Carpina Reservoir (Pernambuco, Brazil)^80^, Lake Tanganyika (Central Africa)^81^, Lake Victoria (Kenya)^82^, Dongping Lake (China)^83^, and Nui Coc Reservoir (Vietnam)^84^. The significant abundance of *cyanobacteria* in all seasons reported in this study suggests that these strains have the ability to adapt to the changing environmental circumstances of reservoirs. The fact that the *cyanobacteria* dominated in summer and could reach higher concentrations than the *cyanobacteria* that dominated in spring and winter indicates greater eutrophication. The increase in *cyanobacteria* can have both positive and negative ecological implications. On the one hand, they contribute to primary production and are a fundamental component of the aquatic food web^85^. On the other hand, some *cyanobacteria* can produce toxins that are harmful to aquatic life and human health, leading to issues when they become overly abundant. Our findings suggest that the proliferation of *cyanobacteria* in the Billings Reservoir can be traced to a confluence of environmental and ecological variables. These blue-green algae are remarkably adaptable to a spectrum of aquatic habitats due to their photosynthetic nature, enabling them to flourish under diverse light conditions and nutrient availabilities, thus outcompeting other species in numerous ecosystems. Notably, the study highlighted a surge in key nutrients such as sulfate, phosphate, ammonium nitrogen, and nitrate nitrogen during November 2019 and January 2020, which *cyanobacteria* efficiently utilize, particularly phosphate, to thrive in eutrophic conditions^86^. Moreover, the optimal median water temperature of 26.85 °C recorded in January bolsters their metabolic activities, accelerating growth and proliferation. Additionally, the reduction in water depth during the dry season presumably enhances light penetration, a boon for the photosynthesis-dependent *cyanobacteria*^79^. The consistent dissolved oxygen (DO) levels observed by the study imply that oxygen availability was not a constraining factor, and the photosynthetic activity of *cyanobacteria* could have contributed to maintaining these stable DO concentrations^87^. The proliferation of *cyanobacteria* leads to the frequent occurrence of algal blooms^88^. During algal blooms, *cyanobacteria* release various low molecular weight compounds such as glucose, organic acids, amino acids, and sugar alcohols, benefiting bacteria in the phycosphere^89^. Additionally, the proliferation and decomposition of algae not only elevate the pH of water but also promote microbial growth, resulting in an increased presence of certain microbial groups in eutrophic waters^90^. However, it is crucial to acknowledge that the abundance of *cyanobacteria* in inland waters, including the observed higher concentrations in the reservoir during summer, is primarily attributed to nutrient loading from agricultural fertilizers, particularly phosphorus, in the catchment area. The decomposition of harmful algal blooms can also release toxins and negatively impact microbial species diversity^91^. Acknowledging the predominant role of fertilizer-driven eutrophication in cyanobacterial dominance is essential for a comprehensive understanding of these blooms^92^. In our study, *Planktothrix* was the numerically dominant bacterial genus in the reservoir in the July and September 2019 samples, while *Microcystis* was the most abundant bacterial species detected in the November and January 2020 samples. Changes in cyanobacterial community structure may be influenced in large part by nutrients in aquatic ecosystems. For example, abundances of *Synechococcus* and *Cyanobium* in East Fork Lake and Delaware Lake were comparable to those in other oligotrophic freshwater lakes^93,94^. According to a study of *cyanobacteria* in Lake Erie, *Synechococcus* and *Cyanobium* were absent for the previous two decades but have since become the dominant taxa as a result of the transition from eutrophic to oligo-mesotrophic conditions^95^. On the other hand, *Planktothrix* has been identified as a bacterial biomarker for lakes that are either eutrophic or hypertrophic^80^. Recently, Zhang et al.^96^ found that *Planktothrix* may live symbiotically with antibiotic-resistant bacteria and secrete antifungal chemicals to promote the emergence and spread of these bacteria. Thus, the detection of *Planktothrix* in the reservoir has far-reaching human health implications beyond conventional cyanotoxin risks.

The second most abundant phylum in the reservoir was *Proteobacteria*. This phylum was indicated as the most abundant phylum in sediments or soils because of its ability to adapt to a variety of toxic conditions and its involvement in organic matter degradation and metabolic processes in lake sediments^97^.

The overall function of the microbial population in surface water of the Billings reservoir increased as eutrophication progressed. Eutrophication had the greatest impact on metabolic functions such as biosynthesis of secondary metabolites and microbial metabolism in different environments. This finding is in agreement with the results of Wan et al.^98^, who studied the diversity of the bacterioplankton population in Lake Nanhu before and after dredging and identified several metabolic pathways.

## Conclusions

The MPA data obtained in this study provide a detailed description of seasonal changes in the taxonomic composition and quantitative ratios of various bacterial groups in the surface waters of Billings Reservoir. The most abundant OTUs belong to the phyla *cyanobacteria* and *proteobacteria*, which constitute a major proportion of each community; then follow the phyla *Actinobacteria*, *Bacteroidetes*, *Verrucomicrobia, Planctomycetes,* and *Chlorobi*. Bacterial communities found in surface waters during the winter season are dominated by *Planktothrix*, in contrast to those found during the summer season, which are dominated by *Microcystis*, which may be explained by seasonal physical and chemical factors. On the other hand, the seasonal variations in the physico-chemical properties of the surface water of the reservoir create new conditions according to which the concentrations of the various bacterial groups change.

### Supplementary Information

Supplementary Figures.

Supplementary Table 1.

Supplementary Table 2.

## Data Availability

The sequencing data generated during the current study are available in the Zenodo repository (DOI https://doi.org/10.5281/zenodo.8298073).
