# Supplementary material for: Temporal patterns of bacterial communities in the Billings Reservoir system
Source: Sci Rep. 2024 Jan 24;14:2062. doi: 10.1038/s41598-024-52432-6 (PMC10808195; doi:10.1038/s41598-024-52432-6)
Supplement: Supplementary file 1 — Supplementary Figures. [file 41598_2024_52432_MOESM1_ESM.docx]

Supplementary Information for:

**Temporal patterns of bacterial communities in the Billings Reservoir System**

Marta Angela Marcondes^1^, Rodrigo Pessoa^1^, Alberto Jose da Silva Duarte^2^, Patricia Bianca Clissa^3^ & Sabri Saeed Sanabani^4^

*^1^Post-Graduation Program in Translational Medicine, Department of Medicine, Federal University of Sao Paulo, So Paulo 04021-001, Brazil.*

*^2^Laboratory of Dermatology and Immunodeficiency, Department of Dermatology LIM 56, Faculty of Medicine, University of Sao Paulo, Sao Paulo 05403-000, Brazil.*

*^3^Laboratory of Immunopathology, Butantan Institute, Sao Paulo 05503-900, Brazil.*

*^4^Laboratory of Medical Investigation 03 (LIM03), Clinics Hospital, Faculty of Medicine, University of Sao Paulo, Sao Paulo 05403-000, Brazil.*

* Corresponding author

Sabri Saeed Sanabani, PhD

E-mail: sabyem_63@yahoo.com

Laboratory of Dermatology and Immunodeficiency, LIM56/03.

Sao Paulo Institute of Tropical Medicine

Sao Paulo Brazil 05403 000

Phone: + 5511 3061 7194 ext:218

Supplementary Figures


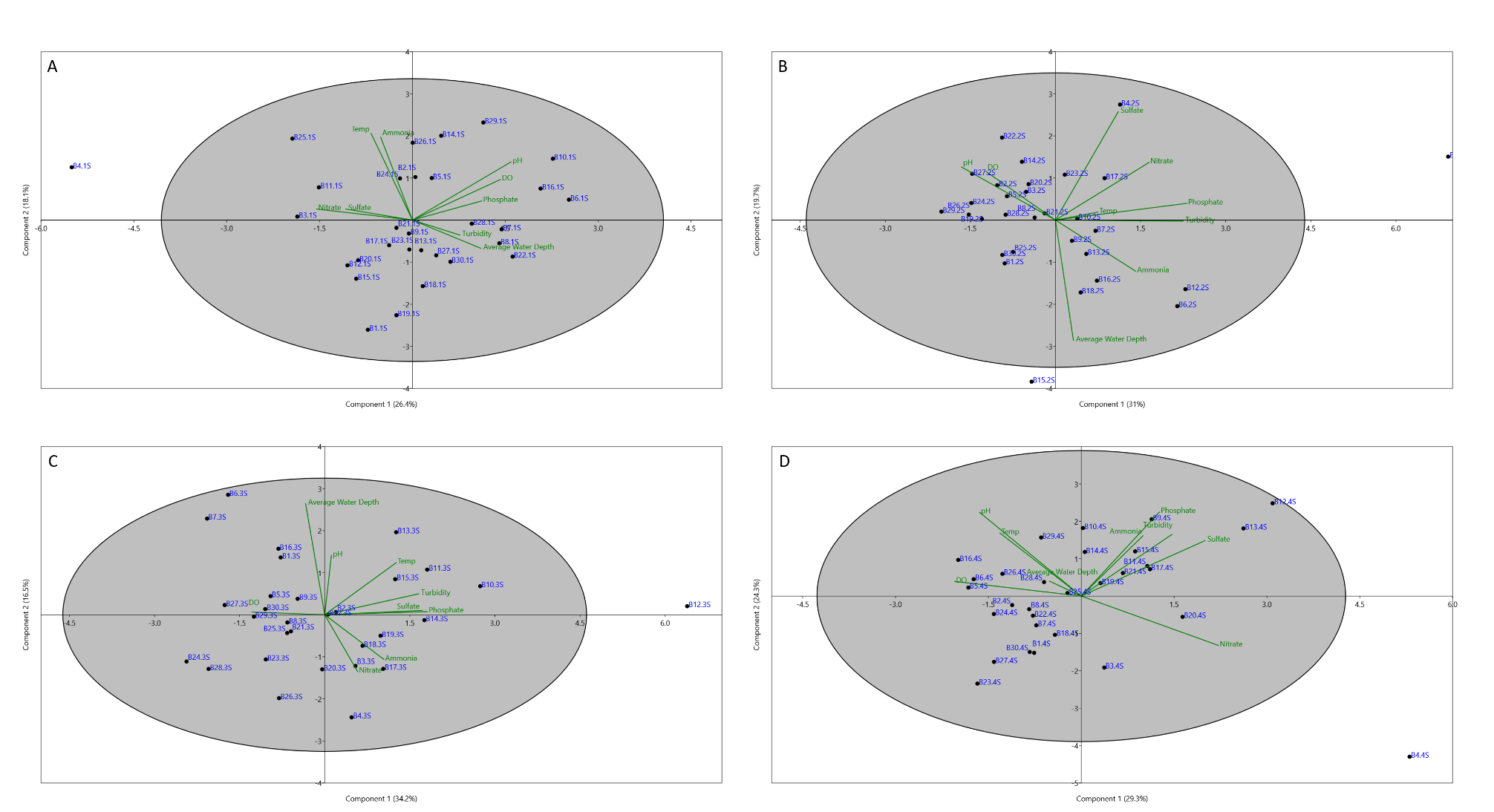


Figure S1. Principal component analysis (PCA) of physicochemical parameters of bimonthly surface water from Billing Reservoir used in this study. Vector lengths indicate the strength of correlation of each parameter with the samples. Black circles represent samples from different sampling periods (A) Samples collected in July 2019 (B) Samples collected in September 2019 (C) Samples collected in November 2019 (D) Samples collected in January 2020.


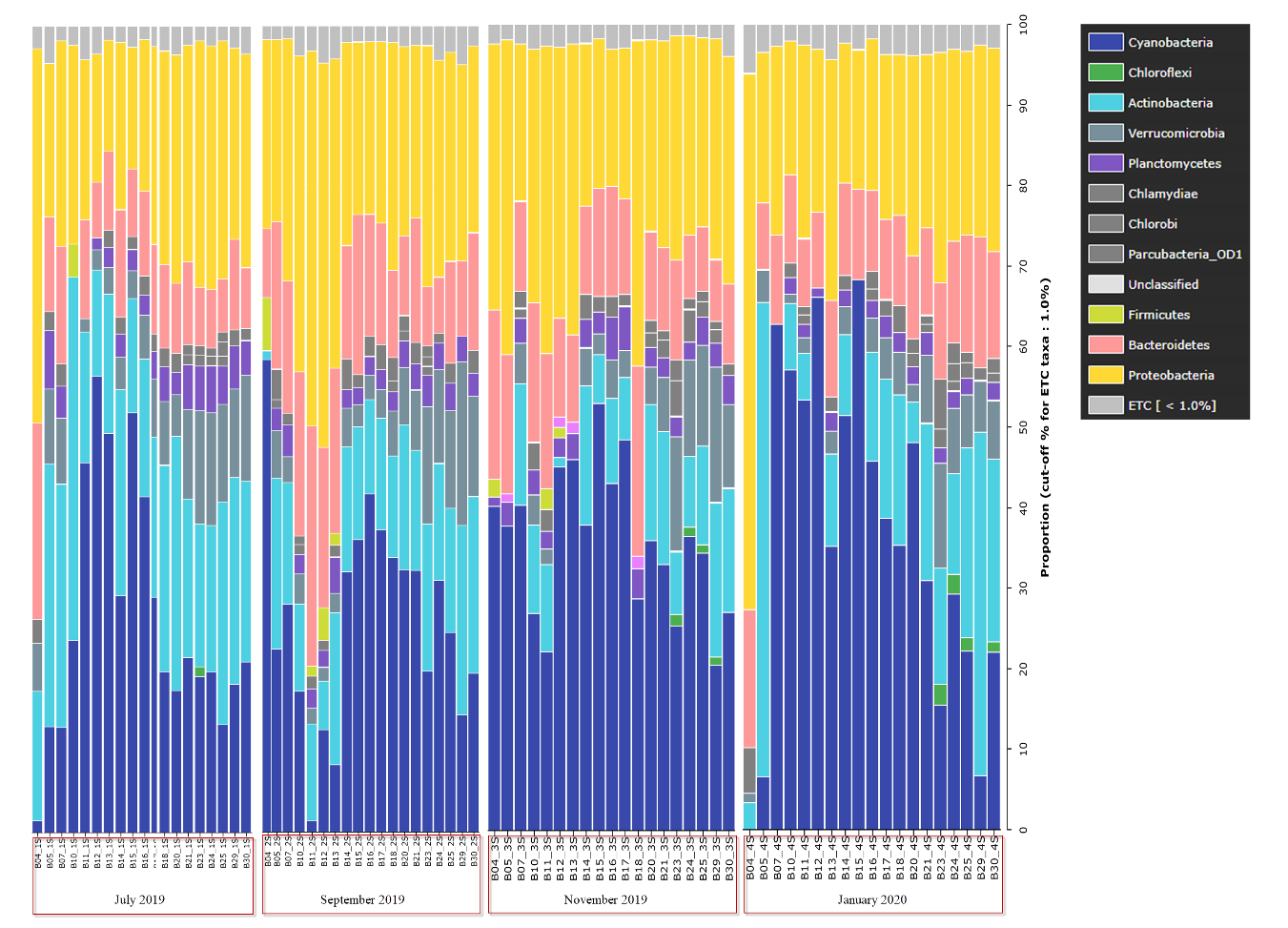


Figure S2. Relative abundance of predominant taxa at the phylum level in bimonthly surface water samples from Billings Reservoir. The legend shows the most abundant phyla in these samples. The height of the color bar indicates the percentage of each taxon in the bacterioplankton community. The x-axis shows the different time periods as indicated in the red empty box.


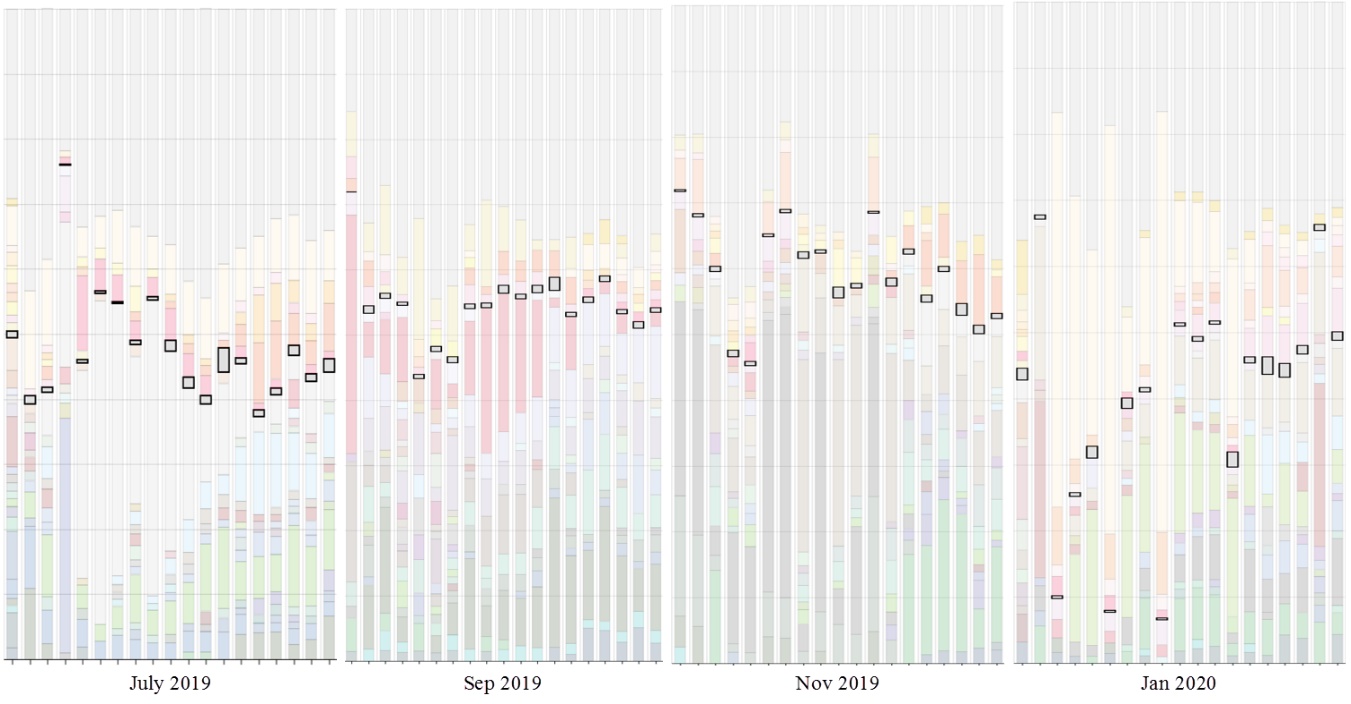


Figure S3. Proportion of unclassified bacterioplankton (indicated by blank black boxes) at the genus level relative to classified genera in the bimonthly surface water of Billings Reservoir.

Supplementary Tables

Table S1. Differentially abundant taxa (minimum LDA score: 2.0) identified by LEfSe analysis in bi-monthly surface water samples collected from Billings Reservoir

Table S2. Functional biomarkers calculated by LEfSe analysis.
